# Melittin Reprograms Tumor‐Associated Macrophages Through CD18‐Mediated Immunomodulation

**DOI:** 10.1002/ptr.70431

**Published:** 2026-08-20

**Authors:** Ik‐Hwan Han, Hyunju Lee, Eunbin Ko, Ilseob Choi, Soyoung Kim, Deok‐Sang Hwang, Hyunsu Bae

**Affiliations:** ^1^ Department of Physiology, College of Korean Medicine Kyung Hee University Seoul Republic of Korea; ^2^ Department of Clinical Korean Medicine, Graduate School Kyung Hee University Seoul Republic of Korea; ^3^ Twinpig Biolab Inc. R&D Center Seoul Republic of Korea

**Keywords:** CD18, immunomodulation, lung carcinoma, melittin, tumor‐associated macrophages

## Abstract

Tumor‐associated macrophages (TAMs), particularly the M2‐like subtype, contribute to an immunosuppressive tumor microenvironment and promote tumor progression. Melittin, a natural bioactive peptide derived from bee venom, has been reported to exert anticancer and immunomodulatory effects; however, the cellular targets and molecular mechanisms underlying its selective action on TAMs remain incompletely defined. This study aimed to investigate the immunomodulatory effects of melittin on TAM subsets and to identify the molecular determinant responsible for its selectivity toward M2‐like macrophages. Macrophage subsets were analyzed following melittin treatment using flow cytometry, gene expression analysis, and immunofluorescence. Binding interactions were assessed by biochemical and biophysical approaches. Tumor growth and immune cell composition were examined in wild‐type and CD18‐deficient mice. Melittin treatment selectively reduced M2‐like TAM populations while increasing infiltration of CD8^+^ cytotoxic T cells in tumor tissues. Mechanistic analyses revealed that melittin preferentially binds to the integrin subunit CD18 (ITGB2), which is enriched in M2‐polarized macrophages, leading to disruption of M2‐associated phenotypes in vitro. Notably, genetic deletion of CD18 abolished the M2‐suppressive and antitumor effects of melittin in vivo. These findings demonstrate that CD18 is functionally required for the immunomodulatory and antitumor activities of native full‐length melittin. This study provides mechanistic insight into the selective action of a natural bioactive peptide and supports its potential as a lead compound for natural product‐based immunomodulatory strategies.

## Graphical abstract

Melittin selectively targets CD18 to deplete M2‐TAMs and remodel the TME to suppress tumor growth.

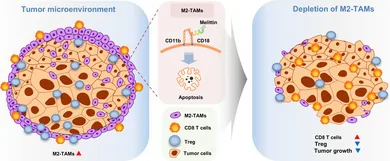

**Abbreviations**
ARG1: arginase 1
CD18: integrin beta 2
CTLA‐4: cytotoxic T‐lymphocyte–associated protein 4
Foxp3: forkhead box P3
ICB: immune checkpoint blockade
ITGB2: integrin beta 2
LLC: Lewis lung cancer
NOS2: nitric oxide synthase 2
NSCLC: non‐small cell lung cancer
PBS: phosphate‐buffered saline
PCNA: proliferating cell nuclear antigen
PD‐1: programmed cell death protein 1
PD‐L1: programmed death‐ligand 1
TAMs: tumor‐associated macrophages
TME: tumor microenvironment
TNF‐α: tumor necrosis factor alpha
Tregs: regulatory T cells

## 1 Introduction

The tumor microenvironment (TME) plays a critical role in tumor progression, metastasis, and therapeutic resistance (Quail and Joyce 2013). In addition to malignant cells, the TME comprises diverse stromal and immune components that dynamically interact to shape cancer development and immune responses. Among these components, tumor‐associated macrophages (TAMs) represent one of the most abundant immune cell populations in solid tumors and have emerged as key regulators of tumor‐promoting inflammation and immune suppression (Mantovani and Sica 2010; Noy and Pollard 2014).

TAMs exhibit functional plasticity and are commonly classified into pro‐inflammatory, tumor‐suppressive M1‐like macrophages and anti‐inflammatory, tumor‐supportive M2‐like macrophages (Noy and Pollard 2014). While M1‐like macrophages contribute to antitumor immunity through the production of inflammatory cytokines and cytotoxic mediators, M2‐like TAMs promote tumor growth by enhancing angiogenesis, extracellular matrix remodeling, and suppression of effector T‐cell activity (Mantovani et al. 2022). In non‐small cell lung cancer (NSCLC), multiple clinical studies have demonstrated that increased infiltration of M2‐like TAMs is associated with poor prognosis, whereas the presence of M1‐like macrophages correlates with improved patient survival (Jackute et al. 2018; Cao et al. 2019). These findings highlight M2‐like TAMs as an attractive therapeutic target for modulating the immunosuppressive TME.

CD18, also known as integrin β_2_ (ITGB2), is a leukocyte‐specific adhesion molecule that forms heterodimers with several α integrin subunits and plays an essential role in immune cell adhesion, migration, and signaling (Dib 2000; Fagerholm et al. 2019). Recent studies have revealed that CD18 expression is preferentially elevated in M2‐like TAMs compared with other macrophage subsets and that high ITGB2 expression is associated with unfavorable clinical outcomes in several cancer types, including lung cancer (Xu et al. 2022; Zu et al. 2022). Importantly, our previous study demonstrated that a melittin‐derived peptide targeting activated CD18 selectively deplete M2‐like TAMs and suppressed solid tumor progression in preclinical models, suggesting that CD18 represents a functionally relevant molecular determinant for TAM‐directed immunomodulation (Han et al. 2025). Therefore, targeting CD18 represents a potentially important therapeutic strategy to modulate M2‐like TAMs‐driven immunosuppression within the TME.

Melittin, a 26‐amino‐acid amphipathic peptide derived from honeybee venom, has been extensively investigated for its antimicrobial, anti‐inflammatory, and anticancer activities (Eisenberg 1984; Carpena et al. 2020; Rady et al. 2017). At high concentrations, melittin disrupts lipid membranes and induces cytotoxicity; however, accumulating evidence indicates that at controlled doses, melittin can exert selective biological effects on immune cells within the TME (Liu et al. 2018). Notably, previous studies have shown that melittin preferentially targets and reduces M2‐like TAMs in the TME, thereby suppressing tumor progression (Lee et al. 2017). More recently, a melittin‐derived peptide targeting activated CD18 was shown to preferentially deplete M2‐like TAMs and suppress solid tumor progression (Han et al. 2025). However, whether native full‐length melittin itself directly interacts with CD18 and whether CD18 mediates its preferential effects on M2‐like TAMs remain incompletely understood.

The aim of this study was to investigate whether melittin selectively targets M2‐like TAMs to induce apoptosis, and to determine whether this effect is mediated through CD18. Thus, these findings could provide a novel strategy to employ therapeutic agents, which target M2‐like TAMs through CD18 in NSCLC.

## 2 Materials and Methods

### 2.1 Animals and Cells

BALB/c wild‐type (WT) mice and CD18 deficient mice (C.129S7(B6)‐Itgb2^tm2Bay^/Abhmj, 8–10 weeks old) were purchased from DBL (Chungbuk, Korea) and Jackson Laboratory. The animal procedures were approved by the University of Kyung Hee Institutional Animal Care and Usage Committee (KHUASP(SE)‐22‐482). All animals were maintained in a pathogen‐free environment on a 12 h light/dark cycle and had access to food and water. The murine Lewis lung carcinoma (LLC) cell line was cultured in Dulbecco's Modified Eagle's medium (DMEM; Welgene, Gyeongsan, Korea) supplemented with 10% fetal bovine serum (FBS; Welgene), 100 U/mL penicillin, and 100 μg/mL streptomycin (Gibco; Thermo Fisher Scientific Inc., Waltham, MA, USA). The human leukemia monocytic cell line (THP‐1) was maintained in RPMI1640 (Welgene) containing 10% fetal bovine serum (FBS; Welgene), 100 U/mL penicillin, and 100 μg/mL streptomycin (Gibco). The cells were cultured every 2–3 days until the cells became 80% confluence, and the cells were incubated at 37°C with 95% humidity and 5% CO_2_ for all experiments.

### 2.2 Macrophage Polarization

THP‐1 monocytes were treated with 100 nM phorbol 12‐myristate 13‐acetate (PMA; Sigma‐Aldrich, St. Louis, MO, USA) for 24 h. The cells were incubated with RPMI1640 containing 10% FBS for 72 h for non‐polarized M0 cells. To generate polarized phenotypes, cells were incubated for 72 h with 20 ng/mL recombinant human interferon (rhIFN)‐γ (Prospec, Ness Ziona, Israel) and with 100 ng/mL lipopolysaccharide (LPS; Sigma‐Aldrich) to obtain M1 macrophages; the cells were incubated with 20 ng/mL recombinant human interleukin (rhIL)‐4 (Prospec) and 20 ng/mL rhIL‐13 (Prospec) to obtain M2 macrophages.

### 2.3 CCK‐8 Assay

To examine the cytotoxic effect of melittin in THP‐1‐derived macrophages, the THP‐1 cells were seeded at 2 × 10^4^ cells/well in 96‐well plates and differentiated into M0, M1, or M2 macrophages using the aforementioned methods. After the differentiation of macrophages, the cells were pretreated with melittin (10, 1, 0.1, 0.01, 0.001, 0.0001, and 0.00001 μM) for 1 h and cultured in a serum‐free medium for 24 h. Cell death was analyzed using the Cell Counting Kit‐8 (CCK‐8) assay. CCK‐8 reagent (Enzo Life Sciences, Farmingdale, NY, USA) was added to each well at a ratio of 1/10 and the plates were incubated for 2 h. The absorbance was measured at 450 nm using a microplate reader (Molecular Devices, San Jose, CA, USA). Data are expressed as the means ± SEM of three independent experiments.

### 2.4 Primary Cell Culture

Primary bone marrow‐derived macrophages (BMDMs) were obtained from mouse femurs. Bone marrow cells were cultured in complete DMEM supplemented with 50 ng/mL macrophage colony‐stimulating factor (M‐CSF; R&D Systems, Minneapolis, MN, USA) for 5 days to induce differentiation into BMDMs. The differentiated BMDMs were treated with 100 ng/mL lipopolysaccharide (LPS) to induce M1‐like macrophages or with 20 ng/mL IL‐4 and IL‐13 (R&D Systems) for 48 h to induce M2‐like macrophages.

### 2.5 Biotin Pull‐Down Assay

Lysates of M2‐differentiated THP‐1 cells were obtained using a Membrane Protein Extraction Kit (Thermo Fisher Scientific). Cell lysates were incubated with scrambled and melittin peptides (0, 0.001, 0.01, 0.1, 1, and 10 μg) and then with biotinylated melittin for 1 h at 4°C with shaking. After incubation, the peptide‐bound membrane proteins were eluted using streptavidin Dynabeads (Thermo Fisher Scientific). Eluted proteins were loaded onto a 10% sodium dodecyl sulfate‐polyacrylamide gel and transferred onto polyvinylidene difluoride membranes. The membrane was blocked and incubated with an anti‐CD18 antibody (LSbio) overnight at 4°C. After washing with TBS containing 25 mM Tris‐Cl, 150 mM NaCl, and 0.05% Tween‐20, the membranes were incubated with anti‐rabbit horseradish peroxidase‐conjugated secondary antibodies. Protein bands were visualized using a Western Blotting Detection Reagent Kit (Thermo Fisher Scientific) and analyzed using ImageJ software (NIH, Bethesda, Maryland, USA).

### 2.6 LC–MS/MS Proteomics Assay

The M0, M1, and M2‐differentiated THP‐1 macrophages were lysed by the Mem‐PER Plus Membrane Protein Extraction Kit (Thermo Fisher Scientific, Waltham, MA, USA). Cell lysates were incubated with biotinylated melittin at room temperature (RT) for 1 h. The mixture was incubated with streptavidin agarose resin (Thermo Fisher Scientific) for 30 min at room temperature. After incubation, the resin was washed. And then binding membrane proteins eluted from the resin. The extracted protein was identified and characterized using LC–MS/MS and proteomics analysis by performing at ebiogen Inc. (Seoul, Korea).

### 2.7 Surface Plasmon Resonance

The binding affinity of melittin and anti‐CD18 antibody to total CD18 protein was measured using surface plasmon resonance (SPR) on a Biacore T200 system (GE Healthcare, Freiburg, Germany). Before the experiment, the surface of the series S sensor chip SA was cleaned with injections of 1 M NaCl and 50 mM NaOH solutions, three times for 1 min each. CD18 protein was immobilized on the SA chip surface at a flow rate of 10 μL/min. The surface was subsequently washed with 50% isopropanol, 1 M NaCl, and 50 mM NaOH to remove non‐specific binding. For the analyte binding studies, anti‐CD18 antibodies and melittin peptide were prepared at concentrations achieving saturated responses below 150 response units (RU). Each analyte was then serially diluted (twofold) four to five times. The multicycle analysis method was used to inject different concentrations of analytes at a flow rate of 30 μL/min to measure binding kinetics. Kinetic data were analyzed using BIA evaluation software (V.3.2.1, GE Healthcare), and the equilibrium dissociation constant (KD) was calculated as KD = *k*_d_/*k*_a_, where *k*_a_ is the association rate constant and *k*_d_ is the dissociation rate constant. Confidence in the results was validated by *U*‐values (< 15) for all analyses.

### 2.8 Animal Study

For tumor model generation, the LLC cells were mixed with Matrigel (Corning, NY, USA), and male BALB/c wild‐type and CD18 deficient mice (8–10 weeks old) were inoculated subcutaneously with 1 × 10^6^ cells per mouse in the right flank (*n* = 4–5/group). Five mice per group were used for the wild‐type model, whereas four mice per group were used for the CD18 deficient model. Melittin (GenScript Corporation, Peace Catterway, NJ, USA) (200 nmol/kg) was administered every 3 days subcutaneously, 7 days after tumor inoculation. Tumor size was examined with a digital caliper every 3 days, and tumor volume was calculated using the formula V = (Width (2) × Length)/2. According to the guidelines, mice were killed when the tumor size attained a maximum diameter of 1–1.5 cm after inoculation. All animal studies were performed with the approval of the Institutional Animal Cared and Use Committee of Kyung Hee University; KHSASP(SE)‐21‐291. All animals were maintained in a specific pathogen‐free environment on a 12 h light/dark cycle with free access to food and water. Nesting sheets were used for enrichment. After the experiments were finished, all mice were euthanized using isoflurane and cervical dislocation.

### 2.9 Flow Cytometry Analysis

To test the affinity of melittin for M2 macrophages, the THP‐1‐derived macrophages that were differentiated into M0, M1, and M2 macrophages were incubated with 100 nM melittin‐FITC (GenScript Corporation, Peace Catterway, NJ, USA) for 1 h at 37°C.

To generate single cells, tumor tissues were chopped into thin pieces and dissociated in DNase I (1 U/mL) (Roche, Indianapolis, IN, USA) and collagenase D (1 mg/mL) (Roche) for 30 min at 37°C with a shaking incubator. The tissues were gently dissociated using the MACS dissociator and the MACS C tube (Miltenyi Biotec, Auburn, CA, USA). The tissues were separated using a 100 μm nylon mesh strainer, and the red blood cells were lysed with BD Pharm lyse buffer (BD Biosciences, San Jose, CA, USA). The single cells were then stained with the following antibodies: mouse CD45‐FITC, CD11b‐PerCP‐Cy5.5, F4/80‐BV421, CD86‐PE‐Cy7, CD206‐APC, CD45‐APC‐Cy7, CD3‐FITC, CD8‐PE‐Cy7, CD4‐PerCP, and CD25‐BV421. All data were detected with a FACS Lyric System (BD Bioscience, CA, USA) and analyzed with FlowJo software (BD Bioscience, OR, USA). The representative gating strategy used to identify tumor‐infiltration T cells and TAMs subsets is shown in Figure S5.

### 2.10 Western Blot Analysis

THP‐1 cells were differentiated into M0, M1, or M2 macrophages using the methods described above. The cells were obtained and lysed in PRO‐PREP protein extraction solution (iNtRON, Bio Inc., Seongnam, Korea). The protein concentrations were measured using the Pierce BCA protein assay kits (Thermo Fisher Scientific, Waltham, MA, USA). Proteins were separated on a 10% sodium dodecyl sulfate‐polyacrylamide gel and transferred to a polyvinylidene difluoride (PVDF) membrane using a Trans‐Blot Turbo Transfer System (Bio‐Rad). The membranes were blocked with 5% bovine serum albumin (BSA) and incubated overnight at 4°C with the following primary antibodies: anti‐cleaved caspase 3 (Cell Signaling Technology) and anti‐β‐actin (1:1000; Abcam, Cambridge, MA, USA). Goat anti‐mouse horseradish peroxidase‐conjugated IgG (Abcam) was used as a secondary antibody. Protein bands were visualized with a D‐Plus ECL Femto System (Donginbiotech Co. Ltd., Korea) and analyzed using ImageJ software.

### 2.11 Quantitative Real‐Time PCR

Total RNA was extracted from cells and mouse tumor tissues using the easy‐BLUE RNA extraction kit (iNtRON Biotechnology, Seongnam, Korea), and the cDNA synthesis was performed using the Maxime RT PreMix Kit (iNtRON Biotechnology), according to the manufacturer's instructions. Quantitative real‐time PCR was performed using the SensiFAST SYBR No‐Rox kit (Bioline, London, UK). The cDNA synthesis conditions were as follows: cycling conditions were 95°C for 15 s, 55°C for 10 s, and 72°C for 10 s. Each reaction was performed in triplicate. Relative mRNA expression was calculated using the 2^−ΔΔCt^ method. Base sequences of primers were listed in Tables S1 and S2. Human‐(CASP3/8/9, ITGB2), mouse‐(NOS2, TNF‐a, Arg‐1, CD206).

### 2.12 Immunofluorescence Analysis and Immunohistochemistry

THP‐1 cells were seeded at 5 × 10^4^ cells/well on chamber slide 4 well (Thermo Fisher Scientific, Waltham, MA, USA), and were polarized into M0, M1, and M2 macrophages using the methods described above. The cells were treated with melittin to assess cell death, or melittin‐FITC (100 nM) for 1 h at 37°C to evaluate binding affinity. For caspase‐3 detection, cells were treated with CellEvent Caspase‐3/7 Green Detection Reagents (Thermo Fisher Scientific, Waltham, MA, USA) (8 μM) during melittin treatment. The cells were washed, fixed with 4% paraformaldehyde for 10 min at room temperature, and cover glasses were mounted in Vectashield mounting medium (Vector Laboratories, Burlingame, CA, USA) with 4′,6‐diamidino‐2‐phenylindole (DAPI) to visualize the nuclei. All images were photographed using the ZEISS LSM 800 laser scanning microscope (Carl Zeiss, Jena, Germany).

Tumor tissues were fixed overnight in 10% neutral buffered formalin and cut to 5 μm with a regular thickness after being embedded in paraffin. The slides were deparaffinized in xylene and rehydrated in ethanol and deionized water. For antigen retrieval, slides were incubated in sodium citrate buffer (pH 6.0) for 15 min in a microwave. The slides were then incubated with a peroxidase‐blocking solution (Dako, Glostrup, Denmark) for 15 min and blocked with 5% bovine serum albumin in PBST (PBS with 0.2% Triton X‐100) for 1 h. Slides were incubated with Arginase‐1 (100:1; Santa Cruz Biotechnology Inc.) or CD18 (100:1; ABclonal) primary antibodies overnight at 4°C, and then visualized with Alexa Fluor 488‐conjugated goat anti‐rabbit (1:500; Invitrogen, CA, USA) or Alexa Fluor 594‐conjugated donkey anti‐goat secondary antibodies (1:500; Invitrogen). Each slide was mounted in Vectashield mounting medium (Vector Laboratories, Burlingame, CA, USA) with DAPI to visualize the nuclei. The tissues were examined using the ZEISS LSM 800 laser scanning microscope (Carl Zeiss, Jena, Germany).

For histological analysis, the tumor sections were incubated overnight at 4°C with PCNA (500:1; Santa Cruz Biotechnology Inc.) or CD8 (500:1; Abcam) or Foxp3 (200:1; ABclonal) rabbit antibody diluted with 5% BSA in PBST. The following day, the sections were incubated with an HRP‐conjugated goat anti‐rabbit IgG secondary antibody (1:400; Solarbio, Beijing, China) for 1 h at room temperature and developed using DAB substrate (Invitrogen). Sections were then counterstained with hematoxylin and examined under a microscope at 20× magnification in at least three randomly selected fields. All images were acquired using an LSM5 PASCAL microscope (Carl Zeiss, Jena, Germany), and total staining intensity was quantified using ImageJ software (NIH).

### 2.13 Statistics

All data were analyzed using Prism 8.0.1 software (GraphPad Software Inc., San Diego, CA, USA) and are presented as mean ± standard error of the mean (SEM). Normality was assessed using the built‐in normality test in GraphPad Prism. For statistical comparisons, an unpaired two‐tailed Student's *t*‐test was used for two‐group comparisons. For multiple group comparisons, one‐way ANOVA followed by the Tukey post hoc test, or two‐way ANOVA followed by Bonferroni correction, was applied as appropriate. *p*‐value < 0.05 was considered statistically significant.

## 3 Results

### 3.1 Affinity and Cytotoxic Effects of Melittin in THP‐1‐Derived M2 Macrophages

To test whether melittin binds selectively to M2 macrophages, THP‐1 cells were differentiated into M0, M1, or M2 macrophages. FITC‐conjugated melittin was used, and the percentage of FITC‐positive cells was measured by flow cytometry. Melittin was shown to bind to M0, M1, and M2 macrophages at 15.5%, 16.8%, and 57.1%. It was seen that melittin had an increased binding for M2 macrophages than M0 and M1 macrophages (Figure 1A). Consistently, melittin's preferential binding to M2 macrophages compared with M0 and M1 was examined by confocal microscopy (Figure 1B). To determine the induction of M2 macrophage apoptosis by melittin, the THP‐1‐derived macrophages were treated with melittin at concentrations ranging between 0.01 nM and 10 μM. The half‐maximal inhibitory concentrations (IC_50_) of melittin in macrophages were evaluated as 2.563, 1.615, and 0.268 μM in M0, M1, and M2 cells, respectively (Figure 1C). Moreover, the expression of caspase‐3 related to cell apoptosis in macrophages treated with melittin was markedly increased in M2 macrophages (Figure 1D,E; Figure S2). Melittin treatment also increased the mRNA expression levels of caspase family members, caspase‐3, 8, and 9, in M2 macrophages (Figure 1F). Consistent with these findings, melittin‐induced CASP3, CASP8, and CASP9 expression was also greater in M2‐like BMDMs than in M0‐ and M1‐like BMDMs (Figure S3B). These results suggest that melittin preferentially binds to M2 macrophages and is accompanied by enhanced activation of apoptotic signaling pathways in these cells.

**FIGURE 1:**
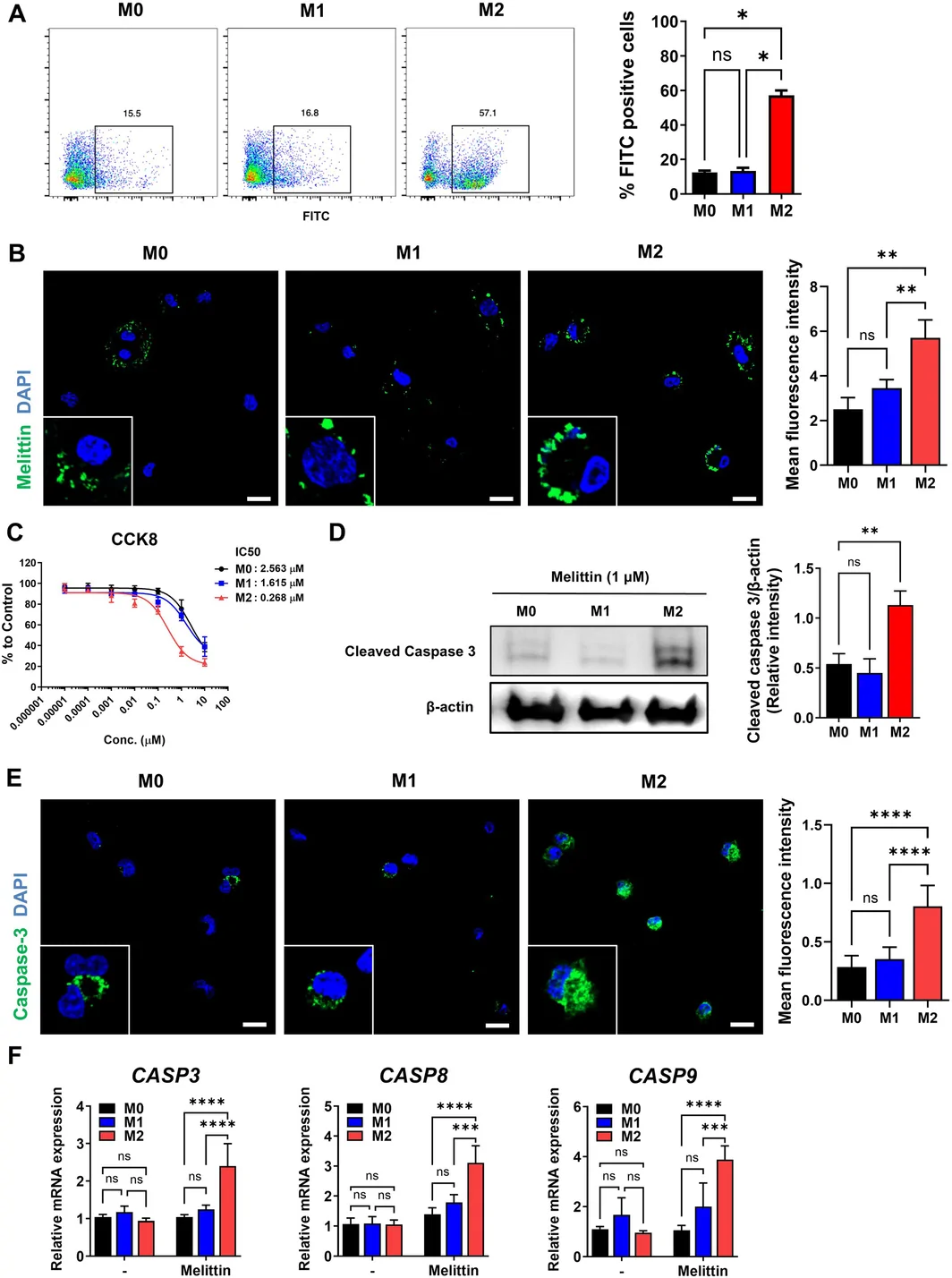
Affinity and cytotoxic effects of melittin in THP‐1‐derived M2 macrophages. (A) Binding affinity of FITC‐conjugated melittin to THP‐1‐derived M0, M1, and M2 macrophages was analyzed by flow cytometry. (B) Preferential association of FITC‐labeled melittin with polarized macrophages was visualized by confocal fluorescence microscopy. (C) Cell viability of THP‐1‐derived macrophages treated with melittin at the indicated concentrations (0.00001–10 μM) was assessed using a CCK‐8 assay. (D) Protein levels of cleaved caspase‐3 in macrophages treated with melittin were analyzed by Western blotting. (E) Activation of caspase‐3 was further examined by immunofluorescence staining. (F) Relative mRNA expression levels of CASP3, CASP8, and CASP9 were quantified by real‐time PCR following melittin treatment. All data are presented as means ± SEM. *p < 0.05, **p < 0.01, ***p < 0.001, ****p < 0.0001.

### 3.2 Identification of ITGB2 as a Target Protein of Melittin Binding to M2 Macrophages

To elucidate the molecular mechanism underlying the preferential interaction of melittin with polarized macrophage subtypes, we performed LC–MS/MS‐based proteomic profiling of melittin‐binding membrane proteins in THP‐1‐derived M0, M1, and M2 macrophages. As a result, ITGB2 (CD18) was among the most consistently enriched membrane proteins in M2 macrophages relative to M0 and M1 populations (Figure 2A). Consistent with these findings, ITGB2 mRNA expression was markedly elevated in M2 macrophages compared with M0 and M1 subtypes (Figure 2B). A similar expression pattern was observed in primary BMDMs, with the highest ITGB2 mRNA expression detected in M2‐like BMDMs (Figure S3A). To determine whether melittin directly interacts with ITGB2, surface plasmon resonance (SPR) analysis was performed using recombinant CD18 protein. Melittin exhibited direct binding to CD18 (KD: 78.6 nM) (Figure 2C). In addition, pull‐down assays using biotinylated melittin demonstrated a concentration‐dependent reduction in ITGB2 capture upon competition with unlabeled melittin, further supporting the specificity of this interaction (Figure 2D). Confocal microscopy analysis revealed prominent colocalization of melittin with ITGB2 at the cell membrane of M2 macrophages (Figure 2E). To assess the functional relevance of ITGB2 in mediating melittin‐induced effects, ITGB2 expression was suppressed using sgRNA‐mediated knockdown (Figure 2F). Reduction of ITGB2 expression significantly attenuated melittin‐induced cytotoxicity in M2 macrophages (Figure 2G) and markedly reduced the induction of apoptotic caspase gene expression (Figure 2H). These results suggest that ITGB2 is preferentially expressed in M2 macrophages and contributes to the selective binding and cytotoxic signaling of melittin.

**FIGURE 2:**
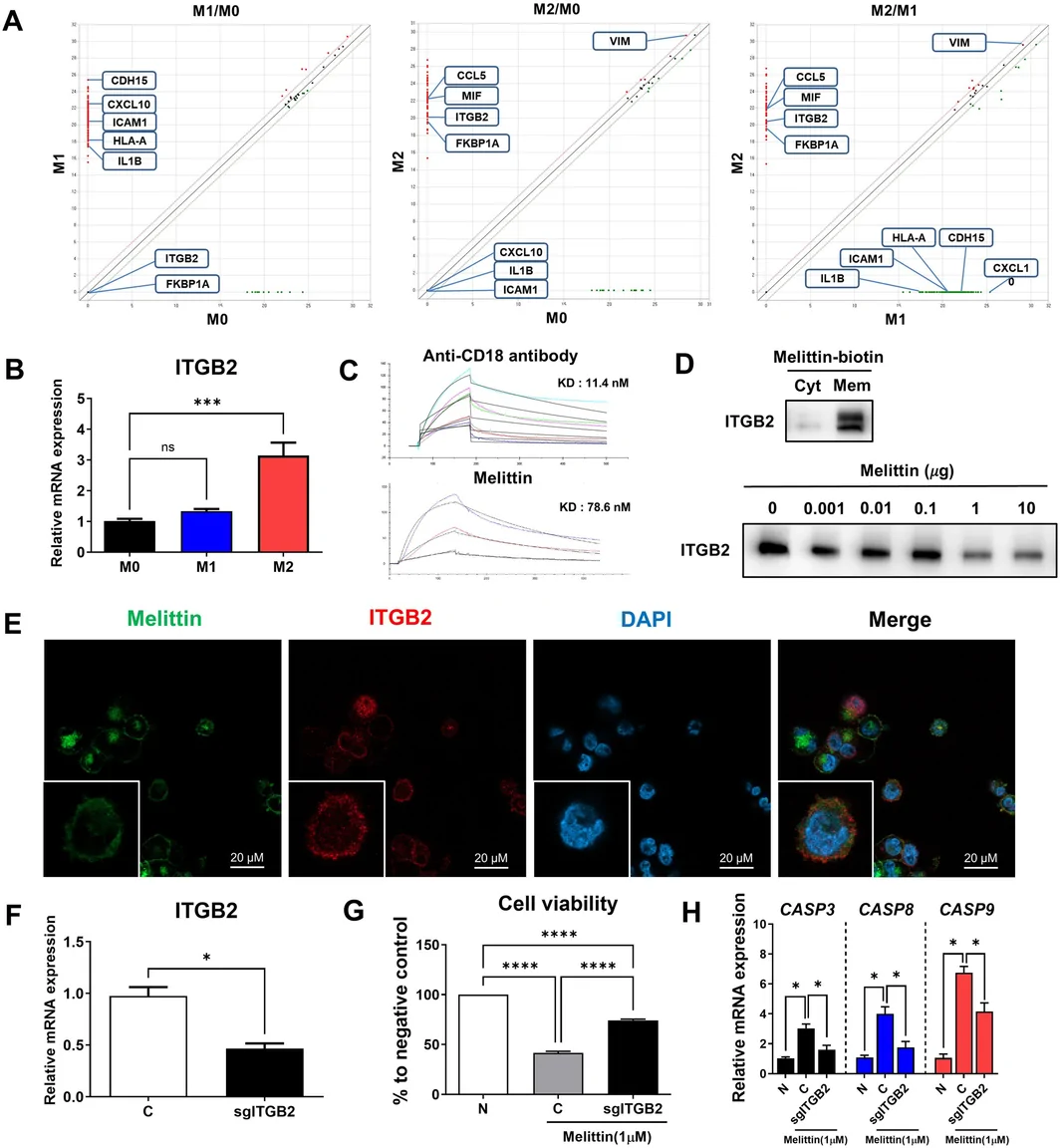
Identification of ITGB2 as a target protein of melittin binding to M2 macrophages. (A) Differential enrichment analysis of melittin‐binding membrane proteins in THP‐1‐derived M0, M1, and M2 macrophages identified ITGB2 as a protein preferentially enriched in M2 macrophages. (B) Relative mRNA expression levels of ITGB2 in THP‐1‐derived M0, M1, and M2 macrophages were quantified by real‐time PCR. (C) Direct interaction between melittin and ITGB2 protein was assessed by surface plasmon resonance (SPR) analysis, and binding kinetics were calculated as indicated. (D) Competitive pull‐down assay showing that increasing concentrations of unlabeled melittin reduce the binding of biotinylated melittin to ITGB2 in M2 macrophage lysates, followed by immunoblot analysis of ITGB2. (E) Colocalization of melittin (green) and ITGB2 (red) in M2 macrophages was visualized by confocal immunofluorescence microscopy. Nuclei were counterstained with DAPI (blue). Scale bar, 20 μm. (F) Relative ITGB2 mRNA expression levels in control and sgITGB2 M2 macrophages were measured by real‐time PCR. (G) Cell viability of control and sgITGB2 M2 macrophages treated with melittin (1 μM) was assessed using a CCK‐8 assay. (H) Relative mRNA expression levels of CASP3, CASP8, and CASP9 in control and sgITGB2 M2 macrophages following melittin treatment (1 μM) were analyzed by real‐time PCR. All data are presented as the mean ± SEM. *p < 0.05, **p < 0.01, ***p < 0.001, ****p < 0.0001.

### 3.3 Suppression of Tumor Growth by Melittin in LLC Model

To evaluate the antitumor efficacy of melittin in vivo, Lewis lung carcinoma (LLC) cells were subcutaneously implanted into BALB/c mice, followed by subcutaneous administration of melittin (200 nmol/kg) or PBS every 3 days starting 1 week after tumor inoculation (Figure 3A). Compared with PBS‐treated controls, melittin‐treated mice exhibited a progressive suppression of tumor growth over the treatment period, as determined by serial measurements of tumor volume (Figure 3B). Significant differences in tumor volume were observed at later time points following melittin administration. Consistently, tumor weight at the experimental endpoint was significantly reduced in the melittin‐treated group relative to the PBS group (Figure 3C). However, no significant differences in body weight were observed between the PBS‐ and melittin‐treated groups during the treatment period (Figure S4A). Histological analysis further demonstrated a marked reduction in the proportion of PCNA‐positive cells in tumor tissues from melittin‐treated mice compared with controls, indicating decreased proliferative activity within the tumor (Figure 3D). These results suggest that melittin effectively suppresses LLC tumor growth in vivo and is associated with reduced tumor cell proliferation.

**FIGURE 3:**
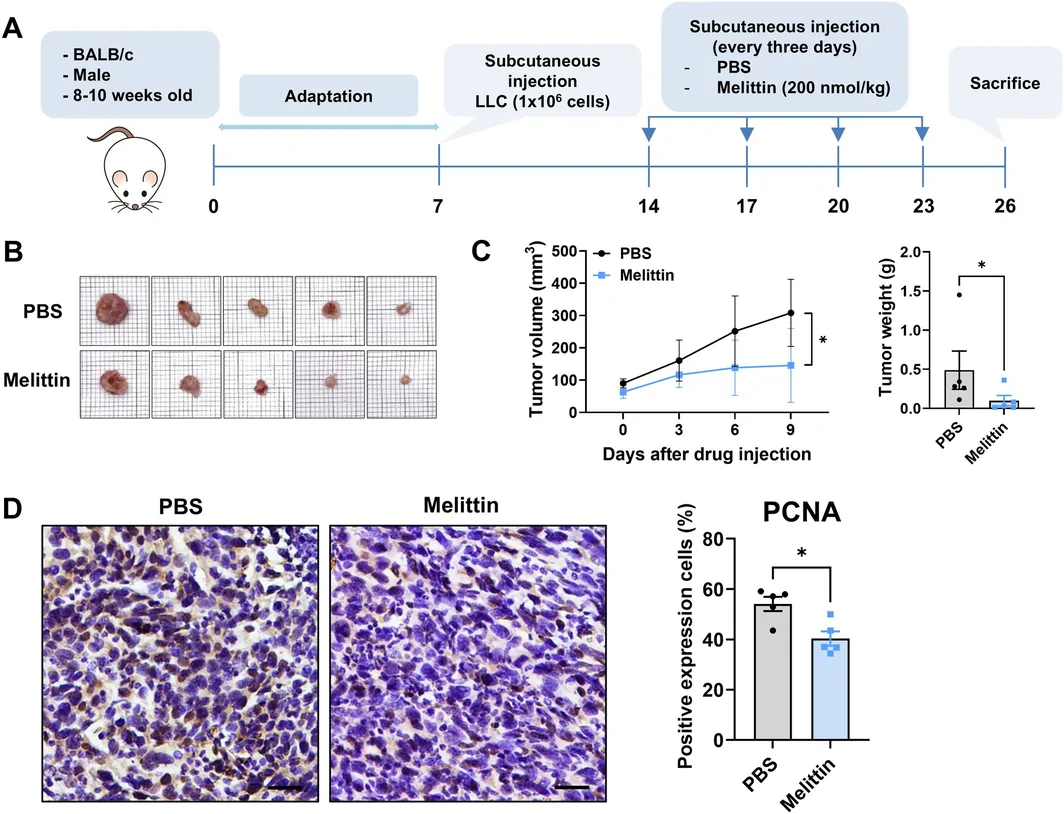
Suppression of tumor growth by melittin in LLC model. (A) Schematic illustration of the in vivo experimental design. BALB/c mice bearing subcutaneous LLC tumors were treated with melittin (200 nmol/kg) or PBS by subcutaneous injection every 3 days. (B) Representative images of excised tumors from PBS‐ and melittin‐treated mice at the experimental endpoint. (C) Tumor growth curves showing tumor volume measured at the indicated time points during treatment (left), and tumor weight measured after sacrifice at the endpoint (right). Tumor volume was calculated using the formula: (width × width × length)/2. (D) Immunohistochemical analysis of proliferating cell nuclear antigen (PCNA) expression in tumor tissues from PBS‐ and melittin‐treated mice. Representative images and quantitative analysis of PCNA‐positive cells are shown. Scale bar, as indicated. Data are presented as mean ± SEM (*n* = 5 per group). *p < 0.05 versus the PBS group.

### 3.4 Reduction of M2‐Like TAMs by Melittin in LLC Model

To determine whether melittin alters the composition of tumor‐associated macrophages (TAMs) within the tumor microenvironment, single‐cell suspensions were prepared from LLC tumors and analyzed by flow cytometry. Melittin treatment resulted in a significant reduction in the proportion of M2‐like TAMs (CD206^+^) among F4/80^+^CD45^+^ cells, whereas the frequency of M1‐like TAMs (CD86^+^) was not significantly changed (Figure 4A). Consequently, the M1/M2 TAM ratio was markedly increased in the melittin‐treated group compared with PBS‐treated controls. Consistent with these findings, further phenotypic analysis revealed that CD206^+^CD18^+^ M2‐like TAMs were significantly reduced following melittin administration, while CD86^+^CD18^+^ M1‐like TAMs remained largely unchanged (Figure 4B). Gene expression analysis of tumor‐infiltrating cells demonstrated increased expression of M1‐associated markers, including *NOS2* and *TNFA*, in the melittin‐treated group (Figure 4C). In contrast, expression of M2‐associated markers such as ARG1 and CD206 was significantly decreased (Figure 4D). Immunofluorescence analysis of tumor sections further confirmed a reduced presence of ARG1‐positive cells in tumors from melittin‐treated mice compared with controls (Figure 4E). In addition, the proportion of CD18‐positive cells within tumor tissues was significantly decreased following melittin treatment (Figure 4F). These data suggest that melittin induces suppression of tumor growth by reducing M2‐like TAMs in a mouse LLC model.

**FIGURE 4:**
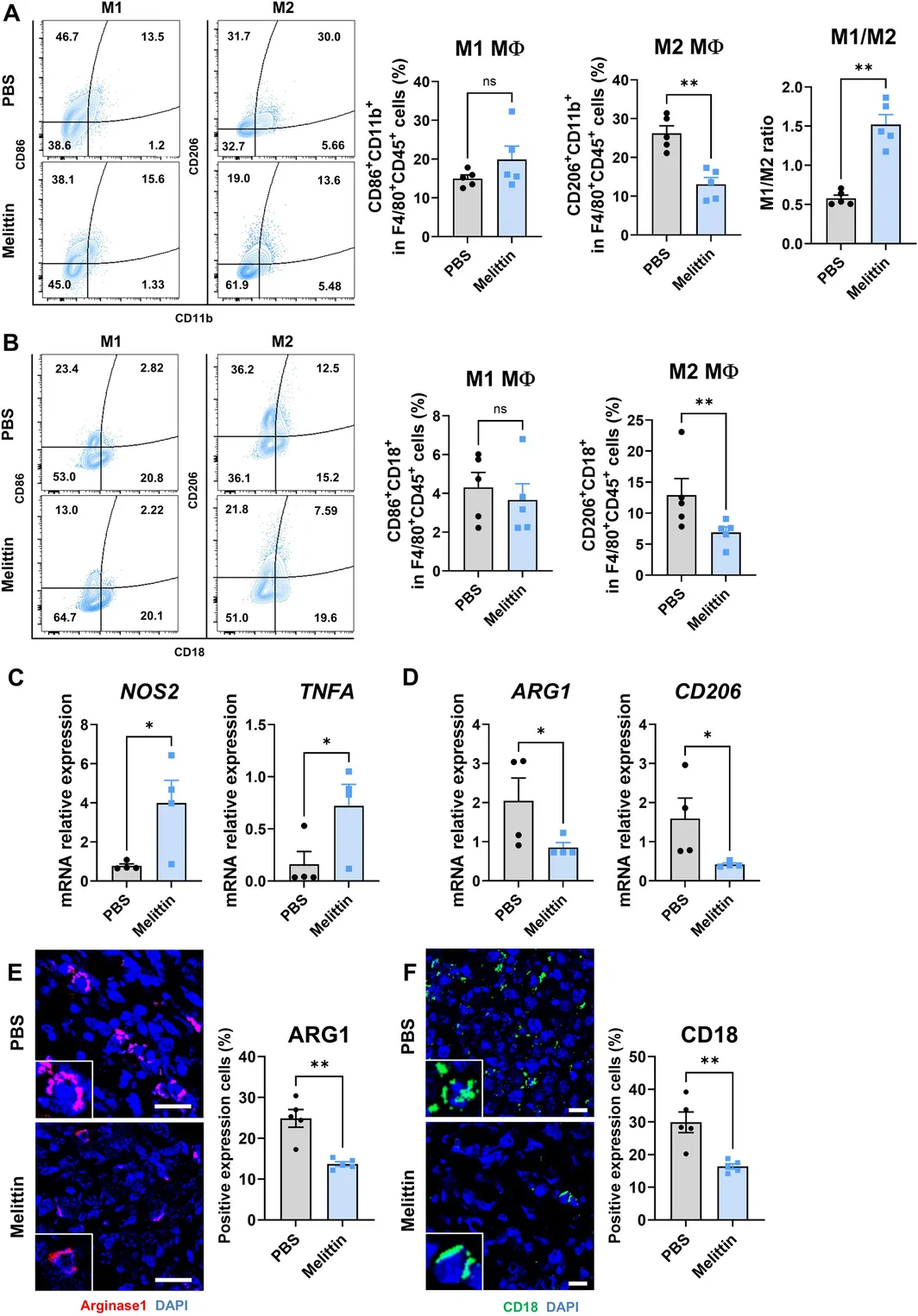
Reduction of M2‐like TAMs by melittin in LLC model. Single cells were harvested from tumor tissues. Single‐cell suspensions were prepared from LLC tumor tissues and analyzed by flow cytometry. (A) Frequencies of M1‐like (CD86^+^) and M2‐like (CD206^+^) tumor‐associated macrophages (TAMs) among CD45^+^F4/80^+^CD11b^+^ cells in PBS‐ and melittin‐treated mice. (B) Flow cytometric analysis of CD18‐expressing TAM subsets showing CD18^+^CD86^+^ M1‐like and CD18^+^CD206^+^ M2‐like macrophages within the CD45^+^F4/80^+^ population. (C, D) Relative mRNA expression levels of M1‐associated markers (NOS2 and TNFA) and M2‐associated markers (ARG1 and CD206) in tumor tissues were quantified by real‐time PCR. (E) Representative immunofluorescence images showing ARG1 expression in tumor sections from PBS‐ and melittin‐treated mice. (F) Representative immunofluorescence images showing CD18 expression in tumor tissues. Scale bars, as indicated. Data are presented as mean ± SEM (*n* = 5 per group). *p < 0.05, **p < 0.01 versus the PBS group.

### 3.5 Alteration of CD8 ^+^ T Cells and Tregs by Melittin in LLC Model

Infiltrated CD8 T cells in the TME play a crucial role in regulating tumor growth (Tang et al. 2024). To determine whether melittin treatment elicited any effect on T cell, the population of CD3^+^CD8^+^ cytotoxic T cells and CD4^+^CD25^+^ regulatory T cells in CD45^+^ cells were measured. Melittin treatment resulted in a significant increase in the proportion of CD3^+^CD8^+^ cytotoxic T cells among CD45^+^ immune cells compared with PBS‐treated controls (Figure 5A). In contrast, the frequency of CD4^+^CD25^+^ regulatory T cells (Tregs) was markedly reduced in tumors from melittin‐treated mice. Consistent with the flow cytometric analysis, immunohistochemical staining of tumor sections revealed a significant increase in CD8‐positive cells in the melittin‐treated group relative to controls (Figure 5B). Conversely, expression of Foxp3, a canonical marker of regulatory T cells, was significantly decreased in tumor tissues from melittin‐treated mice (Figure 5C). Thus, these findings suggest that melittin enhances cytotoxic T cells while reducing immunosuppressive Tregs.

**FIGURE 5:**
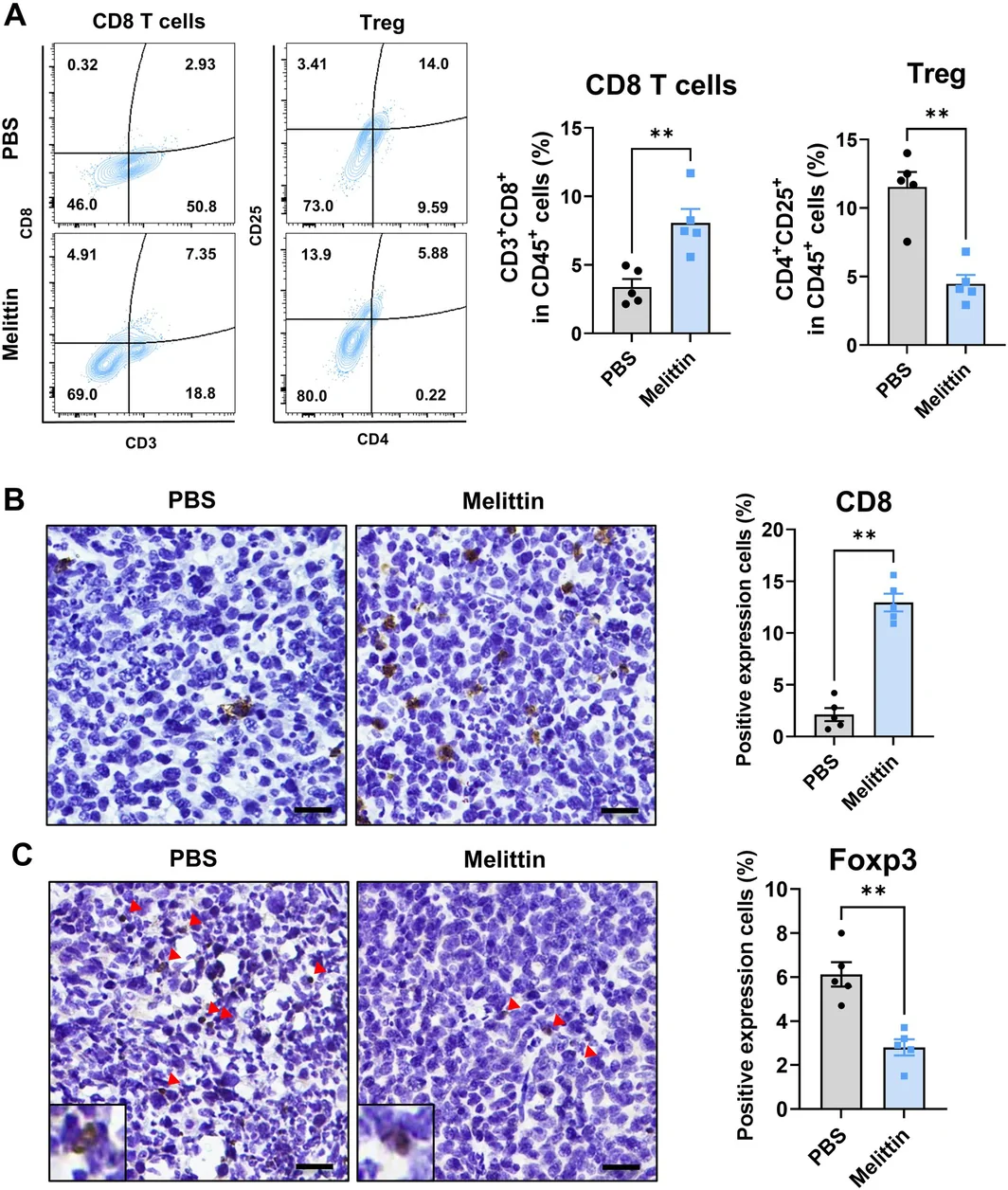
Alteration of CD8^+^ T cells and Tregs by melittin in LLC model. Single‐cell suspensions were prepared from LLC tumor tissues and analyzed by flow cytometry. (A) Representative flow cytometric plots and quantitative analysis showing the frequencies of CD3^+^CD8^+^ cytotoxic T cells and CD4^+^CD25^+^ regulatory T cells (Tregs) among CD45^+^ immune cells in PBS‐ and melittin‐treated mice. (B) Representative immunohistochemical images showing CD8^+^ T‐cell infiltration in tumor tissues from PBS‐ and melittin‐treated mice. (C) Representative immunohistochemical images and quantitative analysis of Foxp3‐positive cells in tumor sections. Data are presented as mean ± SEM (*n* = 5 per group). **p < 0.01.

### 3.6 Abrogation of the Anti‐Tumor Growth Effect of Melittin by CD18 Deficiency

To assess whether the antitumor activity of melittin depends on CD18, Lewis lung carcinoma (LLC) tumor growth was evaluated in CD18‐deficient mice. Loss of CD18 expression in these mice was confirmed prior to tumor implantation (Figure S1). CD18‐deficient mice were subjected to the same tumor implantation and treatment regimen as applied in wild‐type mice (Figure 6A). In contrast to the significant tumor suppression observed in wild‐type animals, melittin treatment failed to reduce tumor growth in CD18‐deficient mice, as indicated by comparable tumor volumes over time and similar tumor weights at the experimental endpoint between melittin‐ and PBS‐treated groups (Figure 6B,C). However, no significant differences in body weight were observed between the PBS‐ and melittin‐treated groups during the treatment period (Figure S4B). Consistently, immunohistochemical analysis of tumor tissues revealed no significant difference in the proportion of PCNA‐positive cells between treatment groups (Figure 6D). These results suggest that CD18 expression is required for the antitumor effects of melittin in the LLC tumor model, providing genetic evidence that CD18 is a key mediator of melittin‐induced tumor suppression.

**FIGURE 6:**
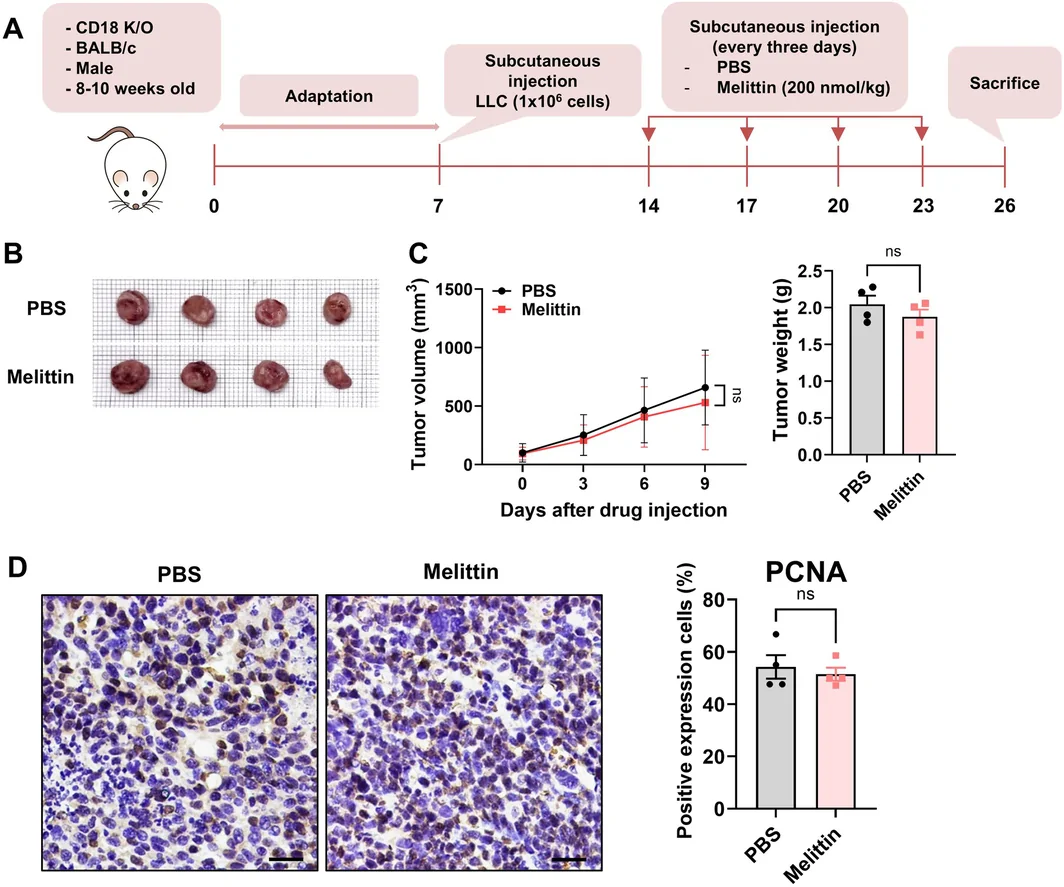
Abrogation of the anti‐tumor growth effect of melittin by CD18 deficiency. (A) Schematic illustration of the experimental design used for CD18‐deficient (KO) mice and wild‐type (WT) controls bearing subcutaneous LLC tumors treated with melittin or PBS. (B) Representative images of excised tumors from WT and CD18 KO mice at the experimental endpoint. (C) Tumor growth curves showing tumor volume measured at the indicated time points during treatment (left) and tumor weight measured after sacrifice at the endpoint (right). Tumor volume was calculated using the formula: (width × width × length)/2. (D) Immunohistochemical analysis of proliferating cell nuclear antigen (PCNA) expression in tumor tissues from WT and CD18 KO mice treated with PBS or melittin. Representative images and quantitative analysis of PCNA‐positive cells are shown. Scale bar, as indicated. Data are presented as mean ± SEM (*n* = 4 per group). ns, not significant.

### 3.7 CD18 Deficiency Abrogated Melittin Mediated Modulation of the TME

To determine whether CD18 is required for melittin‐induced remodeling of the tumor immune microenvironment, immune cell composition was analyzed in LLC tumors derived from CD18‐deficient mice. Flow cytometric analysis revealed no significant differences in the proportions of M1‐like (CD86^+^F4/80^+^) or M2‐like (CD206^+^F4/80^+^) tumor‐associated macrophages (TAMs) among CD45^+^ cells between melittin‐treated and PBS‐treated groups, and the M1/M2 TAM ratio remained unchanged (Figure 7A). Consistently, mRNA expression levels of M1‐associated genes (NOS2 and TNFA) and M2‐associated genes (CD206 and ARG1) were not significantly altered following melittin treatment in CD18‐deficient tumors (Figure 7B,C). Analysis of tumor‐infiltrating T cells further demonstrated no significant changes in the frequencies of CD3^+^CD8^+^ cytotoxic T cells or CD4^+^CD25^+^ regulatory T cells between treatment groups (Figure 7D). In agreement with these findings, immunofluorescence and immunohistochemical analyses showed no significant differences in ARG1 or CD8 expression in tumor tissues from melittin‐treated and control mice (Figure 7E,F). These results demonstrate that CD18 is a critical regulator in the TME, and that melittin loses its antitumor efficacy in the absence of CD18.

**FIGURE 7:**
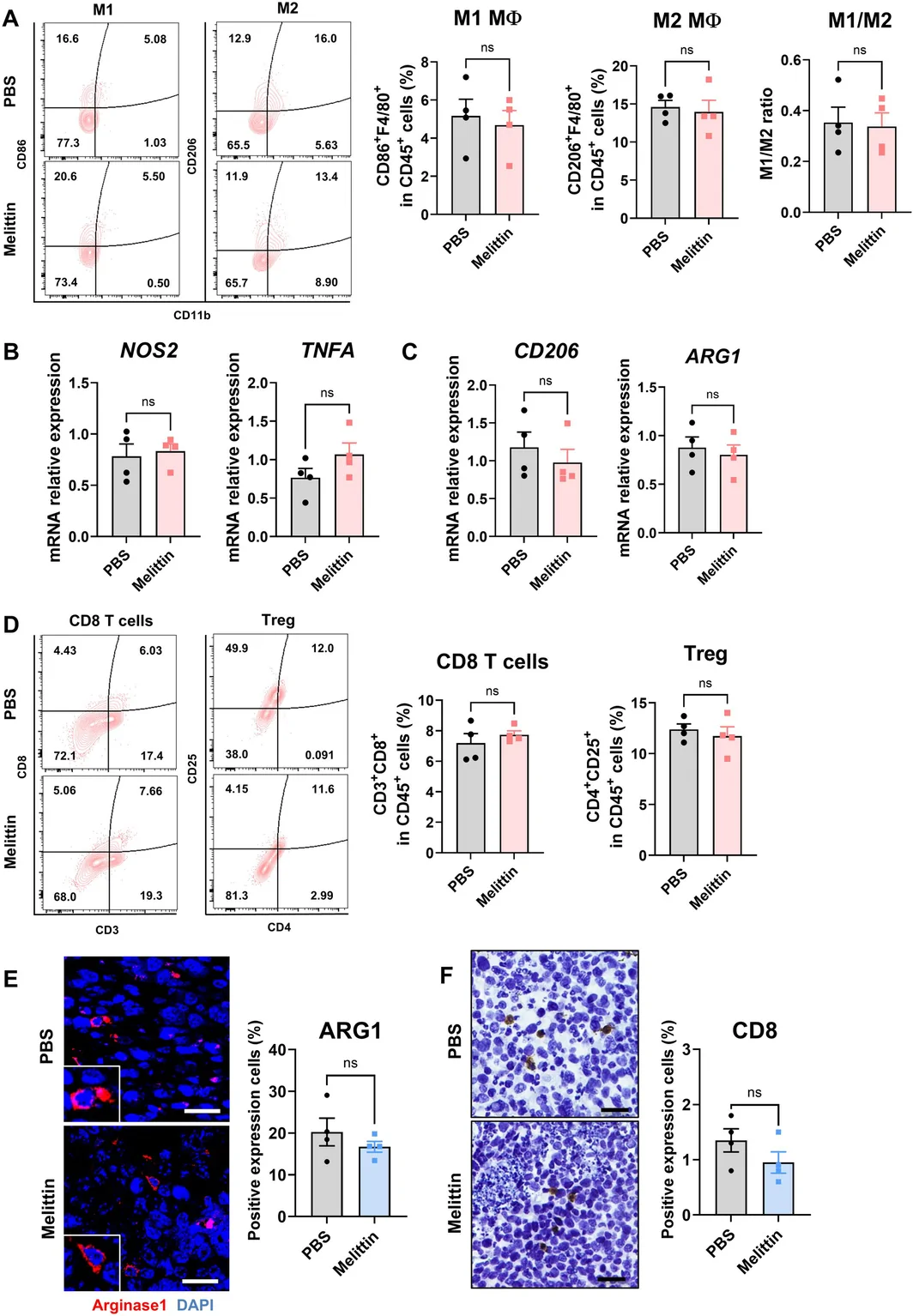
CD18 deficiency abrogated melittin mediated modulation of the TME. Single‐cell suspensions were prepared from LLC tumor tissues of wild‐type (WT) and CD18‐deficient (KO) mice treated with PBS or melittin. (A) Flow cytometric analysis showing the frequencies of M1‐like (CD86^+^) and M2‐like (CD206^+^) tumor‐associated macrophages (TAMs) among CD45^+^F4/80^+^CD11b^+^ cells. (B, C) Relative mRNA expression levels of M1‐associated genes (NOS2 and TNFA) and M2‐associated genes (ARG1 and CD206) in tumor tissues were quantified by real‐time PCR. (D) Flow cytometric analysis of tumor‐infiltrating T‐cell populations showing the frequencies of CD3^+^CD8^+^ cytotoxic T cells and CD4^+^CD25^+^ regulatory T cells (Tregs) among CD45^+^ cells. (E) Representative immunofluorescence images showing ARG1 expression in tumor sections from WT and CD18 KO mice. (F) Representative immunohistochemical images showing CD8^+^ T‐cell infiltration in tumor tissues. Scale bars, as indicated. Data are presented as mean ± SEM (*n* = 4 per group). ns, not significant.

## 4 Discussion

CD18, an integrin β2 subunit predominantly expressed on leukocytes, such as macrophages, plays a crucial role in mediating cell adhesion and migration (Arnaout 1990). Recent studies have further demonstrated that CD18 is preferentially enriched in M2‐like TAMs and that its expression is associated with unfavorable clinical outcomes in several malignancies, including NSCLC (Han et al. 2025; Li et al. 2023; Xu et al. 2022; Zu et al. 2022). These observations suggest that CD18 may serve not only as a prognostic biomarker but also as a functionally relevant molecular determinant within the TME.

Melittin, a major bioactive component of honeybee venom, has long been studied for its antimicrobial, anti‐inflammatory, and anticancer properties (Paull et al. 1977; Jo et al. 2012). While melittin is known to exert nonspecific membrane‐disruptive effects at high concentrations, accumulating evidence indicates that at controlled doses it can elicit selective biological effects on specific immune cell populations. A previous study demonstrated that melittin preferentially associates with M2‐like TAMs and suppresses lung tumor growth in vivo (Lee et al. 2017). However, the molecular basis underlying this apparent selectivity has remained largely undefined. In the present study, we provide mechanistic evidence that CD18 mediates the preferential association of melittin with M2‐like macrophages. Using unbiased proteomic profiling, surface plasmon resonance analysis, and competitive binding assays, we identified CD18 as a direct binding partner of melittin. Functional validation further demonstrated that genetic or sgRNA‐mediated depletion of CD18 markedly attenuated melittin‐induced apoptotic signaling in M2‐like macrophages. Importantly, in CD18‐deficient mice, melittin failed to suppress tumor growth, indicating that CD18 expression is required for the antitumor effects of melittin in vivo. Together, these findings establish CD18 as a critical molecular determinant linking melittin binding to downstream biological outcomes. However, given the broad membrane‐active properties of melittin, additional interactions with other membrane components or CD18‐independent pathways cannot be completely excluded. Further studies are needed to identify potential alternative binding partners of melittin and to determine whether its biological effects are mediated predominantly through CD18 or through interactions with multiple membrane targets.

TAMs represent a dominant immune population within the TME and play a central role in shaping tumor progression and immune evasion (Grivennikov et al. 2010; Wu et al. 2020). In lung cancer, increased M2‐like TAMs has been correlated with poor prognosis (Chen et al. 2003; Takanami et al. 1999). In this study, melittin significantly reduced tumor volume and weight in a murine LLC model and was accompanied by decreased proliferative activity within tumor tissues, as reflected by reduced PCNA expression. Importantly, melittin selectively reduced M2‐like TAM populations, as defined by both CD206‐ and CD18‐expressing subsets, without significantly affecting M1‐like TAMs. Notably, these immunomodulatory effects were completely abolished in CD18‐deficient tumors, underscoring the requirement of CD18 for melittin‐mediated TME remodeling.

Beyond macrophages, effective antitumor immunity critically depends on cytotoxic T‐cell infiltration and the balance between effector and regulatory immune populations. Immune checkpoint blockade (ICB) therapies restore T‐cell function by targeting inhibitory pathways such as PD‐1/PD‐L1 and CTLA‐4, yet their efficacy is often limited by an immunosuppressive TME (Postow et al. 2015; Wei et al. 2018). Strategies that reduce M2‐like TAMs have been shown to enhance CD8^+^ T‐cell infiltration and improve responses to immunotherapy (Cao et al. 2023; Peranzoni et al. 2018). In the present study, melittin treatment in wild‐type mice increased CD8^+^ T‐cell infiltration while reducing regulatory T cells within the TME. Importantly, these effects were absent in CD18‐deficient tumors, indicating that CD18‐dependent modulation of TAMs precedes and likely facilitates subsequent changes in T‐cell composition.

Nevertheless, this study has several limitations. First LLC cells derived from a C57BL/6 background were implanted into BALB/c mice. Although LLC tumors have been successfully established in BALB/c mice in previous studies and in the present study (Chen et al. 2014; Do et al. 2019), the potential influence of strain‐mismatch‐associated immune response on tumor growth and immune cell infiltration cannot be completely excluded. Therefore, further validation using a syngeneic C57BL/6‐background CD18‐deficient model would therefore be valuable.

Second, the pharmacokinetics, biodistribution, bioavailability, and metabolic stability of melittin following subcutaneous administration were not evaluated in this study. Melittin has limited oral bioavailability and a short plasma half‐life and is therefore generally delivered through parenteral routes, including intravenous, intraperitoneal, and subcutaneous injection (Yu et al. 2023). However, the in vivo behavior of melittin following subcutaneous administration remains unclear under the conditions used in this study. Thus, whether the observed effects were mediated primarily by intact melittin, its metabolites, or both remains uncertain. Further studies should compare different administration routes and characterize the circulating and tissue‐associated forms of melittin after administrations.

Third, although CD18 expression was higher in M2 macrophages than in M0 and M1 macrophages, differences in expression levels alone may not fully explain the preferential response of M2 macrophages to melittin. Previous findings have shown that activated CD18 is more abundant in M2‐like TAMs and that an M2‐targeting peptide conjugate preferentially interacts with the cysteine‐rich domain of CD18 in its activated conformation (Han et al. 2025). Therefore, differences in the activation state or conformation of CD18 may have contributed to the preferential effect of melittin on M2 macrophages. However, only total ITGB2 expression was evaluated in this study, and the activation and conformational states of CD18 were not directly compared between M1 and M2 macrophages. Further studies are required to determine whether these factors contribute to the preferential interaction of melittin with M2 macrophages.

## 5 Conclusions

This study identified CD18 as a key target protein mediating the preferential binding of melittin to M2‐like TAMs. We demonstrated that melittin binding to CD18 is required for the induction of apoptotic signaling in M2‐like macrophages, remodeling of the tumor immune microenvironment, and suppression of tumor growth in vivo. By selectively reducing immunosuppressive TAMs and favorably altering T‐cell composition within the TME, melittin emerges as a natural bioactive peptide with distinct immunomodulatory properties. Thus, our findings highlight CD18‐expressing M2‐like TAMs as a promising therapeutic target for natural product‐based strategies aimed at reprogramming the tumor immune microenvironment and providing a mechanistic foundation for future combination approaches with immunotherapy.

## Author Contributions

**Soyoung Kim:** methodology, validation, writing – review and editing, data curation, formal analysis. **Hyunsu Bae:** conceptualization, funding acquisition, writing – review and editing, writing – original draft, project administration, supervision, validation. **Ilseob Choi:** methodology, formal analysis, data curation, writing – review and editing. **Hyunju Lee:** methodology, investigation, data curation, formal analysis, validation, visualization, writing – original draft. **Ik‐Hwan Han:** conceptualization, validation, investigation, writing – original draft, writing – review and editing, visualization, data curation, formal analysis, methodology. **Eunbin Ko:** methodology. **Deok‐Sang Hwang:** methodology, formal analysis.

## Funding

This work was supported by the Ministry of Science and ICT, South Korea and the Korea Institute for Advancement of Technology (RS2024‐00424744).

## Conflicts of Interest

The authors declare no conflicts of interest.

## Supporting information


**Table S1:** qRT‐PCR Human primers for analysis.
**Figure S1:** Validation of CD18 deficiency in tumor‐associated macrophages from CD18 knockout mice. Flow cytometric analysis was performed to assess CD18 expression in M1‐like (CD86^+^) and M2‐like (CD206^+^) macrophages isolated from tumor tissues of wild‐type (WT) and CD18 knockout (KO) mice. Representative flow cytometry plots and quantitative analysis show a near‐complete loss of CD18 expression in both M1‐like and M2‐like macrophage populations from CD18 KO mice compared with WT controls. Data are presented as mean ± SEM (*n* = 4 per group). *p < 0.05, **p < 0.01 versus the WT.
**Figure S2:** Uncropped immunoblots corresponding to Figure 1D.(A) Uncropped Western blot images showing cleaved caspase‐3 protein levels in THP‐1‐derived M0, M1, and M2 macrophages treated with melittin (1 μM). Three independent biological replicates (#1–#3) are shown. Molecular weight markers are indicated on the left, and the position of cleaved caspase‐3 is marked by an arrow. (B) Corresponding uncropped β‐actin immunoblots used as a loading control for the samples shown in (A). The same lane order and biological replicates are maintained. These uncropped images are provided to demonstrate full membrane integrity and loading consistency for the cropped blots presented in Figure 1D.
**Figure S3:** Validation of ITGB2 expression and melittin‐induced apoptotic signaling in primary mouse bone marrow‐derived macrophages (BMDMs). (A) Relative mRNA expression levels of ITGB2 in BMDMs‐derived M0, M1, and M2 macrophages were quantified by real‐time PCR. (B) Relative mRNA expression levels of CASP3, CASP8, and CASP9 in M0, M1, and M2 macrophages following melittin treatment (1 μM) were analyzed by real‐time PCR. All data are presented as the mean ± SEM. *p < 0.05, **p < 0.01, ****p < 0.0001.
**Figure S4:** Body weight changes during melittin treatment. Body weight was monitored in PBS‐ and melittin‐treated wild‐type and CD18‐defecient mice on days 0, 3, 6, and 9 after drug injection. Data are presented as mean ± SEM. ns, not significant.
**Figure S5:** Representative flow cytometric gating strategy for the analysis of tumor‐infiltrating T cells and tumor‐associated macrophage subsets in wild‐type and CD18‐deficient mice. Single‐cell suspensions prepared from LLC tumor tissues of (A) wild‐type (WT) and (B) CD18 knockout (KO) mice were analyzed by flow cytometry. Cellular events were initially selected based on forward‐ and side‐scatter characteristics to exclude debris. Nonviable cells were excluded by Live/Dead staining, followed by singlet discrimination using FSC‐A and FSC‐H. Tumor‐infiltrating leukocytes were identified as CD45^+^ cells. CD8^+^ T cells were defined as CD45^+^CD3^+^CD8^+^ cells, whereas CD4^+^CD25^+^ T cells were identified within the CD45^+^ population. Tumor‐associated macrophages were identified as CD45^+^F4/80^+^CD11b^+^ cells and further classified as CD86^+^ M1‐like or CD206^+^ M2‐like macrophages. CD18 expression was subsequently assessed in the CD86^+^ and CD206^+^ macrophage subsets to confirm CD18 deficiency in tumors from CD18 KO mice. Identical gating boundaries were applied consistently across all experimental groups.

## Data Availability

The data that support the findings of this study are available from the corresponding author upon reasonable request.
