# Supplementary material for: Melittin Reprograms Tumor‐Associated Macrophages Through CD18‐Mediated Immunomodulation
Source: Phytother Res. 2026 Aug 20;40(11):6201–15. doi: 10.1002/ptr.70431 (PMC13753712; doi:10.1002/ptr.70431)
Supplement: Supplementary file 1 [file PTR-40-6201-s001.docx]

**Supplementary Table 1. qRT-PCR Human primers for analysis**

| **Gene** | **F/R** | **Sequence** | **Reference** |
| --- | --- | --- | --- |
| **GAPDH** | **F** | 5’-GGG AGC CAA AAG GGT CAT CA-3′ | NM_001256799.3 |
|  | **R** | 5’-TGA TGG CAT GGA CTG TGG TC-3′ |  |
| **Caspase-3** | **F** | 5’-ACT GGA CTG TGG CAT TGA GA-3’ | NM_001354777.2 |
|  | **R** | 5’-GCA CAA AGC GAC TGG ATG AA-3’ |  |
| **Caspase-8** | **F** | 5’-GCA AAG GAA GCA AGA ACC CA-3’ | NM_001080124.2 |
|  | **R** | 5’-CCT GGT GTC TGA AGT TCC CT-3’ |  |
| **Caspase-9** | **F** | 5’-AAG TGA CCC TCC CAA GTA GC-3’ | NM_001229.5 |
|  | **R** | 5’-GTT CTG GCC AGG TCT CTT CT-3’ |  |
| **ITGB2** | **F** | 5’-ATG TGG ATG AGA GCC GAG AG-3’ | NM_000211.5 |
|  | **R** | 5’-ACT GGG ACT TGA GCT TCT CC-3’ |  |

**Supplementary Table 2. qRT-PCR Mouse primers for analysis**

| **Gene** | **F/R** | **Sequence** | **Reference** |
| --- | --- | --- | --- |
| **GAPDH** | **F** | 5’-GAG AAA CCT GCC AAG TAT GA-3’ | NM_001289726.2 |
|  | **R** | 5’-CTG TTG CTG TAG CCG TAT T-3’ |  |
| **NOS2** | **F** | 5’-CAG CTG GGC TGT ACA AAC CTT-3’ | NM_001313921.1 |
|  | **R** | 5’-CAT TGG AAG TGA AGC GTT TCG-3’ |  |
| **TNF-α** | **F** | 5’-CCA CCA TCA AGG ACT CAA AT-3’ | NM_001278601.1 |
|  | **R** | 5’-CTC AGG GAA TCT GGA AAG-3’ |  |
| **Arg-1** | **F** | 5’-CTC CAA GCC AAA GTC CTT AGA G-3’ | NM_007482.3 |
|  | **R** | 5’-AGG AGC TGT CAT TAG GGA CAT C-3’ |  |
| **CD206** | **F** | 5’-CTC TGT TCA GCT ATT GGA CGC-3’ | NM_008625.2 |
|  | **R** | 5’-CGG AAT TTC TGG GAT TCA GCT TC-3’ |  |

Supplementary Figure 1. Validation of CD18 deficiency in tumor-associated macrophages from CD18 knockout mice. Flow cytometric analysis was performed to assess CD18 expression in M1-like (CD86⁺) and M2-like (CD206⁺) macrophages isolated from tumor tissues of wild-type (WT) and CD18 knockout (KO) mice. Representative flow cytometry plots and quantitative analysis show a near-complete loss of CD18 expression in both M1-like and M2-like macrophage populations from CD18 KO mice compared with WT controls. Data are presented as mean ± SEM (n = 4 per group). ^*^ p < 0.05, ^**^ p < 0.01 versus the WT.

Supplementary Figure 2. Uncropped immunoblots corresponding to Figure 1D.(A) Uncropped Western blot images showing cleaved caspase-3 protein levels in THP-1-derived M0, M1, and M2 macrophages treated with melittin (1 μM). Three independent biological replicates (#1-#3) are shown. Molecular weight markers are indicated on the left, and the position of cleaved caspase-3 is marked by an arrow. (B) Corresponding uncropped β-actin immunoblots used as a loading control for the samples shown in (A). The same lane order and biological replicates are maintained. These uncropped images are provided to demonstrate full membrane integrity and loading consistency for the cropped blots presented in Figure 1D.

Supplementary Figure 3. Validation of ITGB2 expression and melittin-induced apoptotic signaling in primary mouse bone marrow-derived macrophages (BMDMs). (A) Relative mRNA expression levels of ITGB2 in BMDMs-derived M0, M1, and M2 macrophages were quantified by real-time PCR. (B) Relative mRNA expression levels of CASP3, CASP8, and CASP9 in M0, M1, and M2 macrophages following melittin treatment (1 μM) were analyzed by real-time PCR. All data are presented as the mean as ± SEM. *p < 0.05, **p < 0.01, ****p < 0.0001.

Supplementary Figure 4. Body weight changes during melittin treatment. Body weight was monitored in PBS- and melittin-treated wild-type and CD18-defecient mice on days 0, 3, 6, and 9 after drug injection. Data are presented as mean as ± SEM. ns, not significant.

Supplementary Figure 5. Representative flow cytometric gating strategy for the analysis of tumor-infiltrating T cells and tumor-associated macrophage subsets in wild-type and CD18-deficient mice. Single-cell suspensions prepared from LLC tumor tissues of (A) wild-type (WT) and (B) CD18 knockout (KO) mice were analyzed by flow cytometry. Cellular events were initially selected based on forward- and side-scatter characteristics to exclude debris. Nonviable cells were excluded by Live/Dead staining, followed by singlet discrimination using FSC-A and FSC-H. Tumor-infiltrating leukocytes were identified as CD45⁺ cells. CD8⁺ T cells were defined as CD45⁺CD3⁺CD8⁺ cells, whereas CD4⁺CD25⁺ T cells were identified within the CD45⁺ population. Tumor-associated macrophages were identified as CD45⁺F4/80⁺CD11b⁺ cells and further classified as CD86⁺ M1-like or CD206⁺ M2-like macrophages. CD18 expression was subsequently assessed in the CD86⁺ and CD206⁺ macrophage subsets to confirm CD18 deficiency in tumors from CD18 KO mice. Identical gating boundaries were applied consistently across all experimental groups.
